# Before and After: Attitude and Adverse Effects Induced by the First and Second Doses of mRNA BNT162b2 Vaccine among Healthcare Professionals in the First Weeks after Their Introduction in Poland

**DOI:** 10.3390/vaccines11050883

**Published:** 2023-04-22

**Authors:** Gerard Pasternak, Karolina Pieniawska-Śmiech, Mateusz Walkowiak, Joanna Sado, Adam Pytel, Paulina Jasińska, Natalia Kierbiedź-Guzik, Paula Bolaczek, Katarzyna Fleischer-Stępniewska, Mateusz Babicki, Katarzyna Pentoś, Aleksandra Lewandowicz-Uszyńska

**Affiliations:** 1 Third Department and Clinic of Pediatrics, Immunology and Rheumatology of Developmental Age, Wroclaw Medical University, 50-368 Wroclaw, Poland; 2 Department of Immunology and Pediatrics, The J. Gromkowski Provincial Specialist Hospital, 51-149 Wroclaw, Poland; 3 Department of Clinical Immunology, Wroclaw Medical University, 50-368 Wroclaw, Poland; 4 Department of Pulmonology and Pediatrics, The J. Gromkowski Provincial Specialist Hospital, 51-149 Wroclaw, Poland; 5 Department of Infectious Diseases, Liver Diseases and Immune Deficiencies, Wroclaw Medical University, 50-368 Wroclaw, Poland; 6 Department and Faculty of Family Medicine, Wroclaw Medical University, 50-368 Wroclaw, Poland; 7 Institute of Agricultural Engineering, Wroclaw University of Environmental and Life Sciences, 50-368 Wroclaw, Poland

**Keywords:** COVID-19, SARS-CoV-2, vaccine

## Abstract

Background: In the last days of December 2020, the SARS-CoV-2 virus vaccine BNT162b2 (Comirnaty, Pfizer-BioNTech) was introduced, for the first time, for wide use in Poland. According to the vaccination schedule, healthcare workers were the first to receive the vaccine. The aim of this study was to analyse the attitudes of those who were determined to be vaccinated, with particular reference to their concerns, attitudes towards vaccination advocacy and sources of knowledge on vaccination, as well as the incidence of adverse reactions. Methods: The study had a three-stage design. Respondents completed a self-administered questionnaire before receiving the 1st and 2nd vaccine doses and 2 weeks after receiving the 2nd dose. A total of 2247 responses were obtained (1340 responses in the first stage, 769 in the second and 138 in the third). Results: The main source of knowledge on vaccination was the Internet (32%; *n* = 428). Of the respondents, 6% (*n* = 86) reported anxiety before the 1st dose of the vaccine, which increased to 20% (*n* = 157) before the 2nd dose. A declaration of willingness to promote vaccination among their families was made by 87% (*n* = 1165). Among adverse reactions after the 1st dose of the vaccine, respondents most frequently observed pain at the injection site (*n* = 584; 71%), fatigue (*n* = 126; 16%) and malaise (*n* = 86; 11%). The mean duration of symptoms was 2.38 days (SD 1.88). After the 2nd dose of vaccine, similar adverse reactions—pain at the injection site (*n* = 103; 75%), fatigue (*n* = 28; 20%), malaise (*n* = 22; 16%)—predominated among respondents. Those who declared having had a SARS-CoV-2 virus infection (*p* = 0.00484) and with a history of adverse vaccination reactions (*p* = 0.00374) were statistically more likely to observe adverse symptoms after vaccination. Conclusions: Adverse postvaccinal reactions are relatively common after Comirnaty vaccination but are usually mild and transient in nature. It is in the interest of public health to increase the knowledge of vaccine safety.

## 1. Introduction

COVID-19 is a respiratory disease caused by the Severe Acute Respiratory Syndrome Coronavirus 2 (SARS-CoV-2), similar to the other two coronaviruses, SARS-CoV and MERS-CoV. The disease was first described in late December 2019 in China. Due to the increased, progressive spread of infection worldwide, it was declared a pandemic by the World Health Organisation (WHO) in March 2020 [1,2]. At the time of writing this article (late October 2022), the WHO reported more than 620 million confirmed cases of COVID-19 worldwide and 6.5 million associated deaths [3].

Vaccination is undoubtedly a powerful tool to limit the progression and spread of infectious agents. There are two ways to acquire herd immunity: people become infected, or people receive vaccination. The second is undeniably the better option, as the potential harm and risks associated with SARS-CoV-2 infection can be avoided. Vaccination is a very effective method of achieving host immunisation, leading to a coordinated response in terms of innate or acquired immunity, as well as immunological memory.

At the time of writing this article, a total of more than 12 trillion doses of the vaccine have been administered worldwide [4].

Security is the main human need; it concerns not only individuals but also social groups, local communities and citizens of various countries, while the lack of security causes anxiety for individual people and families and can occur on a national and international scale. There are many definitions of health, but the most accurate seems to be the definition according to which health is a state of complete physical, mental and social well-being and not merely the absence of disease or infirmity (introduction to the Constitution of the World Health Organisation) [5].

From the beginning of the pandemic, people literally had to adapt to completely new operating conditions in a matter of days, as restrictions on civil liberties were introduced to limit the spread of the virus. The social life of schools, clubs, restaurants or workplaces became limited to the four walls of homes and computer screens. People were largely deprived of the possibility of direct contact with others, and this is a factor necessary to maintain well-being. 

The epidemiological differences observed in the affected countries underlined the different distribution of risk factors, such as demographics, comorbidities and many other characteristics. In the face of a global pandemic, it is becoming increasingly obvious that the different organisation of health systems and different health policies in terms of health management have a significant impact on the occurrence of a pandemic [6].

The only major infection prevention measures available at the time vaccination was introduced were the use of face masks, social distancing and hand disinfection. Since September 2020, a third, gradual increase in the number of deaths worldwide has been observed. It led to recording the world’s highest daily number of deaths on 21 January 2021 (16,878)—at the same time we conducted our study [7].

In the last days of December 2020, a vaccine against the SARS-CoV-2 virus was introduced for the first time for wide use in Poland. This vaccine was mRNA BNT162b2 (Comirnaty, Pfizer-BioNTech). Comirnaty is an mRNA vaccine containing an informative ribonucleic acid encoding the S (spike) protein of the SARS-CoV-2 virus. Based on the information from the mRNA, the S (spike) protein of SARS-CoV-2 is synthesised in the host cell, which, being a potent antigen, stimulates the response of the immune system in the form of the production of neutralising antibodies (humoral response) and stimulates the production of T lymphocytes (cellular response) [8].

The Polish vaccination schedule prioritised healthcare professionals. The rapid introduction of a new vaccine at a time of a pandemic caused by a pathogen against which there was no effective treatment at the time could certainly have caused concern, including among medical personnel directly involved in the fight for the health and lives of those infected with the coronavirus. Taking into account, among other things, these fears of vaccination and the possibility of the occurrence of hitherto unknown but possible adverse reactions, a 3-stage screening interview questionnaire was prepared for the medical staff vaccinated at the J. Gromkowski Hospital in Wrocław.

The aim of this study was to analyse the attitudes of those who were determined to be vaccinated, with particular reference to their concerns, attitudes towards vaccination advocacy and sources of knowledge on vaccination, as well as the incidence of adverse reactions.

## 2. Materials and Methods

The study was conducted on the basis of a proprietary questionnaire distributed at the COVID-19 vaccination centre of the J. Gromkowski WSS in Wrocław in the period from January to March 2021—those were the first weeks after the introduction of protective vaccinations against SARS-CoV-2 in Poland. The lack of validation of the questionnaires resulted from the fact that the COVID-19 pandemic was a first-time event on a global scale. Therefore, at the time of the introduction of preventive vaccination, there were no studies available that would address issues such as the assessment of knowledge, attitude, acceptance and adverse effects induced by the doses of mRNA BNT162b2 vaccine among healthcare professionals. Similarly to other researchers worldwide, we created questionnaires for this type of analysis ourselves, guided by our own experience and medical knowledge, including regarding supporters and opponents of preventive vaccination. We were curious about the state of knowledge and beliefs of healthcare workers regarding the newly introduced vaccines for use in our country and the world. 

The study was conducted on the basis of the consent of the Bioethics Committee of the Wrocław Medical University, No. KB 152/2021. Participation in the study was completely voluntary and anonymous. All J. Gromkowski hospital employees and healthcare workers from external health units (as well as retired healthcare workers) were invited to participate in the study. Participants in the study were able to resign at any time without giving any reason. Completed questionnaires were collected only from those who gave informed consent for vaccination against SARS-CoV-2 and received the preparation after qualifying for vaccination by a physician. Data were collected using a paper and pencil method where the interviewee completed the form. The author’s knowledge was restricted to the fact that the study’s participants were healthcare professionals who decided to get the vaccine.

The survey was conducted in three stages involving the same study group. Respondents completed forms twice at the point of vaccination before receiving the 1st (Supplementary File S1) and 2nd doses (Supplementary File S2) of the SARS-CoV-2 vaccine, respectively, and a third time (Supplementary File S3) two weeks after completing the vaccination cycle. This vaccine was the only mRNA BNT162b2 (Comirnaty, Pfizer-BioNTech) available at the time. A total of 2247 responses were collected (stage 1—1340, stage 2—769, stage 3—138). 

Statistica 13.0 (TIBCO Software Inc., Palo Alto, CA, USA) and MS Excel 2016 were used to analyse the data. Pearson’s Chi-square test with Yeates’ correction was used to test the statistical correlation between categories of variables. The significance level was established at α = 0.05. A *p*-value less than 0.05 was considered statistically significant. 

## 3. Results

### 3.1. Study Group

The survey received a total of 2247 responses from respondents aged 20–89 years, 1340 responses in the first stage, 769 in the second stage and 138 in the third stage. The mean age was 46.7 years (SD 12.1). The vast majority of respondents were female: 71% in stage one (*n* = 949); 68% in stage two (*n* = 522); and 79% in stage three (*n* = 109). Respondents mainly resided in large cities with more than 500,000 inhabitants (stage 1—64%, *n* = 854; stage 2—69%, *n* = 529; stage 3—75%, *n* = 103). The majority of respondents declared a tertiary education (stage 1—61%, *n* = 816; stage 2—63%, *n* = 481; stage 3—72%, *n* = 99). Healthcare professionals constituted the majority of the respondents, with 1252 people (93%) indicating the above place of employment in stage 1, 685 people (89%) in stage 2 and 134 people (97%) in stage 3. A detailed description of the study group is presented in Table 1.

### 3.2. Fear of Vaccination against SARS-CoV-2

As part of Stage 1 and Stage 2, respondents were asked about their concerns about the aftermath of vaccination. In the period before the first vaccination, anxiety was reported by 86 respondents (6%). Among those prior to the second dose, this percentage increased to 157 (20%). This change is statistically significant (*p* < 0.0005).

Among those reporting anxiety before vaccination, it was significantly more common in women (*p* < 0.0005). No relationship was observed between the prevalence of fears and age, place of residence and education. A detailed description is presented in Table 2.

Those declaring to have had past SARS-CoV2 virus infection were slightly less likely to report fear of vaccination (*p* = 0.03). However, this correlation shows a low value of statistical significance.

Fear of SARS-CoV2 vaccination was significantly more frequent in those not regularly vaccinated against influenza (*p* = 0.00085). For other vaccinations performed in adulthood, an analogous relationship was only observed for pneumococcal vaccination (*p* = 0.017). However, it should be emphasised that the statistical significance is considerably lower for this than in the case of influenza vaccination.

### 3.3. Persuading Family Members to Be Vaccinated against SARS CoV-2

The majority of respondents—as many as 87% (*n* = 1165)—stated that they would urge family members to be vaccinated against COVID-19. The variables analysed showed that residents of large cities (*p* = 0.008), those who regularly vaccinate against influenza (*p* < 0.0005) and those who regularly supplement vitamin D3 were significantly more likely to promote vaccination among their family members. 

A significant relationship occurred for perceived anxiety towards the SARS-CoV-2 vaccination. Respondents feeling anxious about the vaccination were significantly less likely to recommend it among relatives (*p* = 0.00217). A detailed summary is presented in Table 3.

### 3.4. Sources of Knowledge on Vaccination against SARS-CoV-2

As part of the first stage of the survey, respondents were given the opportunity to indicate the sources from which they obtained their knowledge about the SARS-CoV-2 vaccination. Their responses were divided into six categories: internet; television; media (other than television and internet); doctors/experts; information gained through professional work; scientific articles/professional literature; summary of product characteristics (SmPC)/leaflets accompanying the vaccine; other. One person might indicate any number of items. The most common responses included the internet (484 responses—32%), scientific articles/specialised literature (204 responses—15%) and TV programmes (185 responses—14%). No need for information on vaccination was declared by 275 respondents (21%). A detailed summary is presented in Table 4.

### 3.5. Adverse Vaccine Reactions

Among the adverse reactions after the first dose of the vaccine, respondents most frequently observed pain at the injection site (584 cases—71%), fatigue (126 cases—16%) and malaise (86 cases—11%). The mean duration of symptoms was 2.4 days (SD 1.9). 

After the second dose of the vaccine, similar adverse symptoms predominated among respondents and were mainly pain at the injection site (103 cases—75%), fatigue (28 cases—20%), malaise (22 cases—16%) and swelling at the injection site (22 cases—16%). The mean duration of symptoms was 2.9 days (SD 2.3). For both doses, lymph node enlargement lasted the longest (dose 1—6.7 days, dose 2—10.7 days). A detailed summary is presented in Table 5.

Adverse vaccine reactions were observed significantly more often (*p* < 0.0005) by people with secondary (146 people—26%) and higher (359 people—69%) education. A similar statistically significant relationship occurred for individual occupational groups (*p* < 0.0005). Adverse symptoms were significantly more frequently reported by doctors (168 persons—29%), nurses (147 persons—26%) and hospital administration staff (108 persons—19%).

Similar correlations were not observed for factors such as age and place of residence. A detailed summary is presented in Table 6.

### 3.6. SARS-CoV-2 Infection

Those who declared having recovered from SARS-CoV-2 virus infection were statistically more likely to observe adverse symptoms in themselves after vaccination (*p* = 0.00484). This relationship also occurred for specific symptoms such as injection site pain, injection site swelling, fatigue, malaise, chills, fever, muscle and joint pain, headache, nausea/vomiting, lymph node enlargement and insomnia. A detailed summary is presented in Table 7.

### 3.7. Adverse Vaccine Reactions in the Past

In stage 3, respondents were asked about post-vaccination reactions in adulthood. A positive response was marked by 42 people. More than half of the reported adverse reactions occurred after influenza vaccination. 

Those with a history of vaccine reactions were more likely to observe adverse reactions in themselves after SARS-CoV-2 vaccination (*p* = 0.00374). An analogous correlation was found for influenza vaccination (*p* = 0.02053). In the remaining cases, no significant correlation was detected. 

## 4. Discussion

After many months of a pandemic that brought death and suffering while being one of the greatest health challenges of the 21st century, the expectation for the world of science to find an effective cure and/or vaccination was enormous. The approval of vaccines against SARS-CoV-2 and their widespread introduction certainly marked a turning point in the fight against the SARS-CoV-2 pandemic. Paradoxically, when the first vaccine appeared, there were also voices doubting its effectiveness and safety. The level of social fear at that moment of the pandemic was huge, which was confirmed by many studies [9,10]. However, a sufficient percentage of the population needs to be vaccinated to achieve the optimal and expected effect. Hence, it is extremely important to answer the question of how high the level of population immunity against COVID-19 is in Poland, what its components are, and which factors leading to its acquisition are of a modifiable nature. The vaccine distribution system, as well as the promotion of immunisation through vaccination, require effective and population-specific health strategies to ensure high vaccination rates. Nevertheless, the degree of trust in vaccinology professionals, both at the level of the general practitioner and scientific and medical authorities, appears to be important. Given that the main source of knowledge on COVID-19 vaccination, even for healthcare professionals, has been the Internet, it is worth ensuring that the information published there is reliable and scientific and comes from experts in the fields concerned. 

Our classic survey, in the form of a paper questionnaire, at the SARS-CoV-2 vaccination site allowed us to reach a large number of people and a wide age range. The anonymous and voluntary nature of the questionnaire made it possible to obtain information on the percentage of adverse vaccination reactions. Indeed, the authors’ experience shows that the incidence of reporting adverse post vaccinal reactions in Poland, especially among medical personnel, who, in this case, constituted the majority of those surveyed, is relatively low. According to the government data, in the period from 27 December 2020 to 15 February 2022, the percentage of adverse events (AE) after more than 40 million doses of Comirnaty vaccine was 0.017%, of which the majority (0.014%) were mild reactions [11]. In our study, AE-qualifying symptoms were reported by more than 70% of subjects (71% after the first dose vs. 75% after the 2nd dose), but this was also a mild AE—pain at the injection site. Systemic symptoms such as fatigue and malaise were also relatively common (16 and 11% after 1st dose; 20% and 16% after 2nd dose). The discrepancy in these results is most likely related to the complicated and time-consuming procedure for reporting AEs in Poland, which in the case of mild and transient adverse reactions, is ignored in practice. In the study by Lee et al., among Korean healthcare workers who received all three doses of the BNT162b2 mRNA vaccine, systemic adverse reactions were even more common, and they included chills and headache (respectively, 62.6%, 62.4%), followed by myalgia (55.3%), arthralgia (53.4%), fatigue (51.6%), pruritus (38.1%), and fever (36.5%). Similar to our study, the most common local adverse event was injection site pain. It occurred after at least one dose in 98.0% of workers [12].

For comparison, in the online survey from Saudi Arabia, a significant number of patients reported such symptoms as injection site pain (54%), followed by muscle and/or joint pain (36.3%), then fatigue and headache (35.1%) after the first dose of vaccine but it is worth mentioning that in this study, other types of vaccine (Oxford-ChAdOx1 nCoV-19 and mRNA-1273) were also assessed [13]. Another study from Saudi Arabia by Alkhalifah et al., which was conducted among 28,031 individuals, found that 53. 6% of all side-effects were reported following Pfizer-BioNTech. The most prevalent SARS-CoV-2 vaccine side effects were mild in nature. The most common reported AEs after the Pfizer-BioNTech vaccine was fatigue and the frequency of this AE was similar to our study (18.35%) [14].

Allergic reactions were not common in our study (1% after 1st dose; 0% after 2nd dose), and no incidence of anaphylaxis was reported. In the study by Akaishi et al., acute allergic reactions (0.05–0.005%) and anaphylaxis (<0.005%) were also not common after the 3rd and 4th doses of the vaccine [15]. It provides valuable information that acute allergic reactions are a relatively uncommon adverse event after SARS-CoV-2 vaccination, which is confirmed by other studies as well [16,17,18]. However, in the study by Abukhalil et al. in the Birzeit University community, allergic reactions were much more common—reported by 12.7% of participants [19]. The authors classified allergic skin reactions (itching, burning, and rash), angioedema, shortness of breath, coughing, and significant swelling of the tongue or lips as allergic reactions. In case of allergic complications associated with vaccination and especially anaphylactic reactions, there is a superiority of trials based on professional medical assessment rather than self-assessment during questionnaire studies.

It is interesting to note that a declaration of recovery from SARS-CoV-2 virus infection was statistically more frequently associated with adverse reactions after vaccination (*p* = 0.00484), which may be due to immunological mechanisms. In contrast, the higher incidence of AE in individuals with a history of AE, including after influenza vaccination, suggests a personal predisposition.

A study by Nomura et al. investigating reasons for uncertainty about COVID-19 vaccination in the Japanese population found that the most common reason for reluctance or uncertainty about vaccination was anxiety about the COVID-19 vaccine, especially its side effects [20]. Despite the fact that only those who eventually decided to receive the vaccine participated in our study, it is noteworthy that 6% of subjects before the first dose and, surprisingly, as many as 20% of subjects before the second dose experienced vaccine-related anxiety. It may be that the occurrence of AE was the reason for the increased anxiety before receiving the next dose of the vaccine. 

At the time of writing (as of 23 October 2022), when a total of 57,269,433 vaccinations had been performed in Poland (including 22,570,847 fully vaccinated persons), the number of reported AEs was 18,611 [21]. In our study, the number of reported AEs was relatively high, with as many as 71% of respondents experiencing pain at the injection site after the 1st dose of vaccine, with a much smaller proportion reporting systemic symptoms such as fatigue (16%), malaise (11%). The frequency of local AE was slightly higher after the second dose of the vaccine, and the symptoms were of short duration and transient in nature. The proportion of serious AEs was very low, but symptoms found after the 1st dose of the vaccine may have caused more anxiety before the second dose than with the first dose (20% vs. 6%). The incidence of AE was comparable to those described in the multinational, placebo-controlled, observer-blinded, pivotal efficacy trial by Polack et al. in the NEJM, but there the incidence of local AE was lower after the 2nd dose and higher after the first dose [22]. It is noteworthy that the majority of AEs were mild/moderate (mild to moderate) in nature and yet may have contributed to increased anxiety before the second dose. It would therefore be worth considering whether more widespread campaigns explaining the mechanisms of action of vaccines and the induction of the immune response, as well as emphasising the benefit/risk ratio for all vaccines approved for use, would have the effect of reducing the level of vaccine anxiety and therefore a higher proportion of vaccination of the population. 

In the Nomura et al. study [20], the second reason for uncertainty or reluctance to accept vaccination was a general, sceptical attitude towards vaccination (not only against SARS-CoV-2), particularly related to its side effects and overall safety. It is noteworthy that, as in other studies [23,24,25], it has been shown that people who get influenza vaccines, are generally more positive about other vaccinations. Respondents who were unsure whether they would vaccinate against COVID-19 were also more likely not to vaccinate against influenza. In our study, fear of being vaccinated against SARS-CoV2 was significantly more common among those not regularly vaccinated against influenza (*p* = 0.00085). Building confidence in vaccination, in general, is, as can be seen, crucial in the context of wider public health. 

The high percentage of vaccinated individuals among medical personnel (in Poland in July 2021, according to statistics from the Supreme Chamber of Physicians, more than 90% of doctors and 85% of dentists had received two doses of the COVID-19 vaccine) confirms that the awareness of current medical knowledge as well as the complications of the disease is also extremely important factors in raising awareness of the need for disease prevention through active immunisation [26]. Many scientific sources demonstrate that when complex science policy issues become the subject of national or even worldwide debate, the level of education and knowledge are very important tools to help a citizen understand the arguments of supporters and opponents. In the study by Miller et al., education, biological literacy and understanding of the coronavirus were strong positive predictors of the willingness to be vaccinated [27]. In our study, anxiety before vaccination was not correlated with the level of education. However, we should remember that participants of our study had already decided to be vaccinated. Those declaring passage of SARS-CoV-2 virus infection in our study were less likely to report fear of vaccination. Interestingly, also in terms of vaccination advocacy, those who were regularly vaccinated against influenza (*p* < 0.0005) and those who took vitamin D regularly (*p* = 0.00467) were more positive and, therefore, aware of existing prevention guidelines. An online study by Babicki et al. on the Polish population, including medical personnel, women, people living in large cities, and those with higher education presented positive attitudes towards COVID-19 vaccination [28]. In contrast, in our study, among those reporting concerns about vaccination, they were significantly more common in women (*p* < 0.0005). No correlation between the prevalence of fears and age, place of residence and education was observed. In a meta-analysis by Robinson et al., female gender, young age, lower income and/or education level, and belonging to an ethnic minority were associated with lower levels of intention to vaccinate against COVID-19 [29].

The main limitation of the survey was that it was only applicable to those already determined to be vaccinated against SARS-CoV-2 due to the location of the questionnaire and its nature. Another limitation was the unvalidated questionnaire. Assessment of adverse events was based only on subjective participant reporting.

## 5. Conclusions

Understanding the factors that influence acceptance or anxiety about COVID-19 vaccination is very important. The fear of vaccination that results in denial and avoidance of this form of prophylaxis can cause serious health consequences. In the public space, at the time of starting vaccination against SARS-CoV-2, due to their rapid introduction (less than a year of the pandemic), there were many skeptical voices that could result from natural fear and a small number of multi-centre studies on complications after this particular vaccination. It is important to remember the basic principle “prevention is better than cure” and to be aware that the course of COVID-19 can vary widely, including severe, with a high risk of death and, in the case of recovered patients, complications. Immunisation is the safest, most effective and cheapest form of prevention. The fashion for a healthy lifestyle should not be associated with the negation of the undeniable and effective achievements of modern medicine, as vaccination definitely gives us a chance to preserve our health and lives. Education on disease prevention in its broadest sense is also essential among healthcare professionals, as it brings tangible benefits on many levels. It is in the interest of public health to increase knowledge about the safety of vaccination and the benefit/risk ratio of vaccination against SARS-CoV-2. It is certainly warranted to conduct further research on the relationship between risk factors and the occurrence of adverse reactions after any vaccination. The authors believe that understanding fears about vaccination will help to run effective campaigns to encourage vaccination in the future.

Moreover, comparative effectiveness research on different systems would provide precious information to develop better organisational models to face the pandemic. Thus, it is important to share methods and outcomes. Referring to the sphere of operation of the healthcare system, it can be said that public health is the basis of this system by focusing on protection against diseases and threats from the living and working environment, and protection against threats from inappropriate social conditions. Health organisations and governments around the world should focus on developing joint plans to avoid a new emergency without a cure. 

## Figures and Tables

**Table 1:** Characteristics of the study group by stage of the study.

Variable		Stage 1 (*n* = 1340)		Stage 2 (*n* = 769)		Stage 3 (*n* = 138)		*p*
Age	18–29	133	10%	47	6%	15	11%	*p* < 0.001
	30–39	305	23%	146	19%	43	31%	
	40–49	327	24%	240	31%	36	26%	
	50–59	333	25%	238	31%	26	19%	
	>60	242	18%	98	13%	18	13%	
Sex	Male	391	29%	247	32%	29	21%	*p* = 0.025
	Female	949	71%	522	68%	109	79%	
Place of residence	Village	239	18%	119	15%	25	18%	*p* = 0. 004
	<50,000	125	9%	75	10%	6	4%	
	50–100 thousand	35	3%	18	2%	3	2%	
	100–500 thousand	87	6%	28	4%	1	1%	
	>500,000	854	64%	529	69%	103	75%	
Education	Basic	12	1%	10	1%	0	0%	*p* = 0.008
	Medium	428	32%	220	29%	39	28%	
	Professional	84	6%	58	8%	0	0%	
	Higher	816	61%	481	63%	99	72%	
Are they a healthcare worker?	Yes	1252	93%	685	89%	134	97%	*p* < 0.001
	Not	88	7%	84	11%	4	3%	
Chronic diseases	Hypertension	-	-	-	-	19	14%	
	Type 1 diabetes	-	-	-	-	0	0%	
	Type 2 diabetes	-	-	-	-	11	8%	
	Obesity	-	-	-	-	14	10%	
	COPD	-	-	-	-	0	0%	
	Recurrent respiratory tract infections	-	-	-	-	0	0%	
	Bronchial asthma	-	-	-	-	8	6%	
	Heart diseases	-	-	-	-	8	6%	
	Cancer	-	-	-	-	1	1%	
	Other	-	-	-	-	3	2%	

Abbreviations: ‘-’—lack of data.

**Table 2:** Vaccination concerns by age, gender, and socioeconomic information.

Variable		Do They Feel Apprehensive about Being Vaccinated against SARS-CoV-2?				
		Not		Yes		*p*
		*n* = 1866		*n* = 243		
Age	18–29	160	9%	20	8%	*p* = 0.297
	30–39	399	21%	52	21%	
	40–49	497	27%	70	29%	
	50–59	498	27%	73	30%	
	>60	312	17%	28	12%	
Sex	Men	596	32%	42	17%	*p* < 0.001
	Women	1270	68%	201	83%	
Place of residence	Village	320	17%	38	16%	*p* = 0.170
	<50,000	178	10%	22	9%	
	50–100 thousand	42	2%	11	5%	
	100–500 thousand	106	6%	9	4%	
	>500,000	1220	65%	163	67%	
Education	Basic	20	1%	2	1%	*p* = 0.719
	Medium	576	31%	72	30%	
	Professional	129	7%	13	5%	
	Higher	1141	61%	156	64%	
		Have they experienced SARS-CoV-2 infections?				
		Yes	Not	*p*		
Do they feel apprehensive about being vaccinated against SARS-CoV-2?	Not	196	1589	*p* = 0.034		
		10.5%	85.2%			
	Yes	35	194			
		14.4%	79.8%			

**Table 3:** Propagation of SARS-CoV-2 vaccination among family versus variables.

Variables		Are They Going to Persuade the Family?				*p*
		Not		Yes		
		*n* = 175		*n* = 1165		
Place of residence	Village	**14**	**8%**	73	6%	*p* = 0.008
	<50,000	9	5%	26	2%	
	50–100 thousand	25	14%	100	9%	
	100–500 thousand	32	18%	207	18%	
	>500,000	95	54%	759	65%	
Do you regularly vaccinate against influenza?	Not	141	81%	673	58%	*p* < 0.001
	Yes	34	19%	492	42%	
Do they take vitamin D3 regularly?	Not	99	57%	522	45%	*p* = 0.004
	Yes	76	43%	643	55%	
Do they feel apprehensive about being vaccinated against SARS-CoV-2?	Not	154	88%	1100	94%	*p* = 0.002
	Yes	21	12%	65	6%	
Do they own animals?	Not	108	62%	579	50%	*p* = 0.003
	Yes	67	38%	586	50%	
Do they smoke cigarettes?	Not	134	77%	920	79%	*p* = 0.470
	Yes	41	23%	245	21%	
Do they drink alcohol?	Not	47	27%	420	36%	*p* = 0.113
	Yes	12	7%	60	5%	
	Occasional	116	66%	703	60%	

**Table 4:** Declared sources of knowledge on SARS-CoV-2 vaccination.

	Number of People Using the Information Source	%
Internet	428	32%
TV	185	14%
Media	174	13%
Doctors/experts	113	8%
Work	169	13%
Scientific articles/specialised literature	204	15%
SmPC/leaflet	28	2%
Other	49	4%
Does not seek information on vaccination	275	21%

Abbreviations: SmPC—summary of product characteristics.

**Table 5:** Prevalence and duration of individual adverse effects.

	Stage 2			Stage 3		
Reported Adverse Reactions	Prevalence (*n* = 769)		Duration [Days]	Prevalence (*n* = 138)		Duration [Days]
Pain at the injection site	548	71%	1.6	103	75%	2.5
Swelling at the injection site	49	6%	2.2	22	16%	2.6
Redness at the site of injection	36	5%	1.6	20	14%	3.4
Pruritus at the injection site	16	2%	2.3	6	4%	3.7
Fatigue	126	16%	2	28	20%	3.1
Poor well-being	86	11%	1.9	22	16%	3.1
Shivers	51	7%	1.6	7	5%	1.5
Fever	30	4%	1.4	6	4%	1.4
Muscle and joint pain	76	10%	1.8	15	11%	4.7
Headache	62	8%	1.9	7	5%	2.2
Nausea/vomiting	16	2%	6.5	6	4%	1.2
Diarrhoea	6	1%	2.3	2	1%	4.5
Enlargement of lymph nodes	15	2%	6.7	4	3%	10.6
Insomnia	5	1%	5.5	2	1%	3
Acute peripheral facial nerve palsy	1	0%	-	1	1%	1
Allergic reaction	4	1%	1	0	0%	-
Anaphylaxis/anaphylactic shock requiring the administration of adrenaline	0	0%	-	0	0%	-

Abbreviations: ‘-’—lack of data.

**Table 6:** Incidence of adverse reactions after vaccination in different demographic groups.

Variable		Adverse Reactions after Vaccination				
		Yes		Not		*p*
		*n* = 560		*n* = 347		
Age	18–29	40	7%	22	6%	*p* = 0.061
	30–39	126	22%	63	18%	
	40–49	171	30%	105	30%	
	50–59	165	29%	99	29%	
	>60	58	10%	58	17%	
Sex	Male	152	27%	124	36%	*p* = 0.006
	Female	408	72%	223	64%	
Place of residence	Village	91	16%	53	15%	*p* = 0.563
	<50,000	43	8%	38	11%	
	50–100 thousand	13	2%	8	2%	
	100–500 thousand	19	3%	10	3%	
	>500,000	394	69%	238	69%	
Education	Basic	2	0%	8	2%	*p* < 0.001
	Medium	146	26%	113	33%	
	Professional	17	3%	41	12%	
	Higher	395	69%	185	53%	
Profession	Doctor	168	29%	99	29%	*p* < 0.001
	Nurse	147	26%	66	19%	
	Paramedic	11	2%	19	5%	
	Salesperson	19	3%	18	5%	
	clerk	108	19%	71	20%	
	technician	41	7%	44	13%	
	Other	66	12%	30	9%	

**Table 7:** Incidence of adverse reactions after vaccination and its type according to the SARS-CoV-2 outbreak.

		SARS-CoV-2 Infection				*p*
		Yes		No		
		*n*= 140		*n* = 767		
Adverse reactions after vaccination	Not	101	72%	459	60%	0.004
	Yes	39	28%	308	40%	
Type of adverse reactions	Pain	112	80%	539	70%	0.027
	Swelling at the injection site	25	18%	46	6%	<0.001
	Redness at the injection site	13	9%	43	6%	0.148
	Pruritus at the injection site	3	2%	19	2%	0.962
	Fatigue	42	30%	112	15%	<0.001
	Poor well-being	39	28%	69	9%	<0.001
	Shivers	24	17%	34	4%	<0.001
	Fever	18	13%	18	2%	<0.001
	Muscle and joint pain	30	21%	61	8%	<0.001
	Headache	21	15%	48	6%	<0.001
	Nausea/vomiting	9	6%	13	2%	0.002
	Diarrhoea	3	2%	5	1%	0.218
	Enlargement of lymph nodes	11	8%	8	1%	<0.001
	Insomnia	4	3%	3	0%	0.011
	Acute peripheral facial nerve palsy	0	-	2	0%	0.711
	Allergic reaction	2	1%	2	0%	0.224
	Anaphylaxis/anaphylactic shock requiring the administration of adrenaline	0	-	0	-	1.0000

Abbreviations: ‘-’—lack of data.

## Data Availability

The authors confirm that the data supporting the findings of this study are available within the article and its Supplementary Materials.

## Supplementary Materials

The following supporting information can be downloaded at: https://www.mdpi.com/article/10.3390/vaccines11050883/s1; Supplementary File S1: Questionnaire for patients preparing for injection with the mRNA anti-COVID-19 Vaccine; Supplementary File S2: Questionnaire for patient attending dose II of COVID-19 mRNA Vaccination; Supplementary File S3: Questionnaire for patient who has received two doses of mRNA Vaccination against COVID-19.
