# Supplementary material for: Before and After: Attitude and Adverse Effects Induced by the First and Second Doses of mRNA BNT162b2 Vaccine among Healthcare Professionals in the First Weeks after Their Introduction in Poland
Source: Vaccines (Basel). 2023 Apr 22;11(5):883. doi: 10.3390/vaccines11050883 (PMC10222834; doi:10.3390/vaccines11050883)
Supplement: Supplementary file 1 [file vaccines-11-00883-s001.zip › Vaccines-2278513-Supplementary File S1.pdf]

QUESTIONNAIRE FOR PATIENTS PREPARING FOR INJECTION WITH THE mRNA anti-COVID-19 Vaccine

AGE:
GENDER: female, male
Place of residence:

[ ] village
[] city of up to 50,000 inhabitants
[] city of 50,000 to 100,000 inhabitants
[] city of 100,000 to 500,000 inhabitants
[] city of more than 500,000 inhabitants

Number of people living together: .... Number of rooms:................................

Education: ....

Do you have pets at home? What kind if any?……………………………………………….

What is your blood group? ....

Do you smoke cigarettes?

[ ] Yes [ ] No

Do you drink alcohol?

[ ] Yes [ ] No [ ] Occasionally

Are you a health care professional?

[ ] Yes [ ] No

Do you suffer from chronic diseases, such as diabetes, hypertension, heart disease, respiratory diseases (e.g. COPD, asthma), cancer, etc.?

[ ] Yes, ... (please specify)

[] No

Do you take chronic medication?

[ ] Yes, ... (please specify)

[] No

Do you regularly vaccinate against influenza?

[ ] YES

[ ] No (please give reason)..

Do you regularly take vitamins?

[ ] YES, ... (please specify)

[] No

Do you regularly take other nutritional supplements?

[ ] YES, ... (please specify)

[] No

Do you suffer from allergic rhinitis and/or conjunctivitis, bronchial asthma, atopic dermatitis, contact dermatitis, angioedema, acute/chronic urticaria?

[ ] YES, ... (please specify disease)

[] I have symptoms of one or more of the diseases listed, but have not yet been diagnosed

[] Not stated

Have you ever had any adverse vaccination reactions?

[ ] Yes, ... (please specify which)

[] No

Have you ever had an allergic reaction to drugs/contrast agents/food/insect venom/contact agents e.g. dyes?

[ ] YES, ... (please specify)

[] No

Have you ever had an anaphylactic reaction, including anaphylactic shock?

[ ] YES, ... (please specify trigger)

[ ] YES, trigger has not been established

[] NO

Have you ever received adrenaline in your life as part of the treatment for an allergic reaction?

[ ] Yes [ ] No

Do you have a history of SARS-CoV-2 infection?

[ ] YES, confirmed by molecular testing (PCR) or antigen test

[] I had/have typical symptoms of SARS-CoV-2 infection, but without confirmation by laboratory tests

[] I have a positive titer of anti-CoV IgG antibodies and have had symptoms of infection

[] I have a positive titer of anti-CoV IgG antibodies, but have had no symptoms of infection

[] NO

Do you suffer from thrombocytopenia/blood clotting disorders/are you taking anticoagulants?

[ ] Yes [ ] No

Have you been diagnosed with an immune disorder (primary/secondary immunodeficiency) or you are taking immunosuppressive drugs?

[ ] Yes [ ] No

Do you feel apprehensive about being vaccinated against SARS-CoV-2?

[ ] Yes, ... (please specify)

[] No

Where do you get your information on vaccination against SARS-CoV-2 ?

....

Do you urge your family and loved ones to be vaccinated against SARS-CoV-2 ?

[ ] Yes

[] No
