# Supplementary material for: Before and After: Attitude and Adverse Effects Induced by the First and Second Doses of mRNA BNT162b2 Vaccine among Healthcare Professionals in the First Weeks after Their Introduction in Poland
Source: Vaccines (Basel). 2023 Apr 22;11(5):883. doi: 10.3390/vaccines11050883 (PMC10222834; doi:10.3390/vaccines11050883)
Supplement: Supplementary file 1 [file vaccines-11-00883-s001.zip › Vaccines-2278513-Supplementary File S2.pdf]

QUESTIONNAIRE FOR PATIENT ATTENDING **DOSE II OF** COVID-19 mRNA VACCINATION

AGE:
GENDER: female, male
Place of residence:
[ ] village
[ ] city of up to: 50,000 inhabitants [ ], city of 50,000 to 100,000 [ ], city of 100,000 to 500,000 [ ], city of more than 500,000 inhabitants
Number of people living together: .... Number of rooms:..
Education: ....
Are you in active employment? [ ] Yes [ ] No
Occupation: doctor, nurse, paramedic, orderly, administrative worker, technical worker (please tick the appropriate one)
What is your blood group? ....

Do you smoke cigarettes? [ ] Yes [ ] No

Do you drink alcohol? [ ] Yes [ ] No [ ] Occasionally

Do you regularly engage in any sports activities? ....

Do you regularly vaccinate against influenza?
[ ] YES
[ ] No (please state the reason)...

Which diseases/pathogens did you vaccinate against as an adult (please tick the appropriate one)

Hepatitis B Hepatitis A Pneumococcus Meningococcus Tetanus Diphtheria
Other..

Do you regularly take vitamin D3 (in what doses?)?
[ ] YES

[] No

Do you take nutritional supplements?
[ ] YES, ...(please specify which ones)
[] No

Do you suffer from chronic diseases such as, for example, diabetes, hypertension, heart disease, respiratory diseases (e.g. COPD), cancer and others?
[ ] Yes, ... (please specify)
[] No

Do you take chronic medication?
[ ] Yes, ...(please specify)
[] No

Do you have a blood coagulation disorder /myelopoiesis, plasma blemishes, congenital thrombosis/ are you taking anticoagulants?
[ ] Yes [ ] No

Have you been diagnosed with an immune disorder (primary/ secondary immunodeficiency)?
[ ] Yes [ ] No

Are you taking medications with immunosuppressive effects?
[ ] Yes [ ] No

Do you suffer from allergic rhinitis and/or conjunctivitis, bronchial asthma, atopic dermatitis, contact dermatitis, angioedema, acute/chronic urticaria?
[ ] YES, ... (please specify the disease)
[] I have symptoms of one or more of the diseases listed, but have not yet been diagnosed
[] Not found

Have you ever had any adverse vaccination reactions?
[ ] Yes, ... (please specify which ones and after which vaccine)
[] No

Have you ever had a serious allergic reaction to drugs/contrast agents/food/insect venom/contact agents e.g. dyes, heavy metals, latex, rubber?
[ ] YES, ...(please specify)
[] No

Have you ever had an anaphylactic reaction, including anaphylactic shock?
[ ] YES, ... (please specify the triggering factor)
[ ] YES, the triggering factor has not been established
[] NO

Have you ever received adrenaline in your life for the treatment of an allergic reaction?
[ ] Yes [ ] No

Do you have a history of SARS-CoV-2 infection?
[ ] YES, confirmed by molecular testing (PCR) or antigen test
[] I had/have typical symptoms of SARS-CoV-2 infection, but without confirmation by laboratory tests
[] I have a positive titer of anti-CoV IgG antibodies and have had symptoms of infection
[] I have a positive titer of anti-CoV IgG antibodies, but have had no symptoms of infection
[] NO

Did you experience any adverse reactions after receiving the first dose of the vaccine; if so, on which day?
[ ] Yes [ ] No

	Pain at the injection site	Swelling at the injection site	Redness at the injection site	Pruritus at the injection site	Fatigue	Poor well-being	Shivers	Fever	Muscle/joint pain

YES									
NO									
Duration									

	Headache	Nausea	Diarrhoea	Enlargement of lymph nodes	Insomnia	Acute peripheral facial nerve palsy	Allergic reaction	Anaphylaxis/anaphylactic shock requiring adrenaline supply
YES								
NO								
Duration								

Have you had any other symptoms of concern not included above within 7/21 days of the first dose?
[ ] YES, ... (please specify) [ ] NO

Have the symptoms listed above affected your life activities?
[ ] YES [ ] NO [ ] I did not have any adverse reactions after vaccination

Did you experience symptoms of an allergic reaction within 15-30 minutes after the first dose of the vaccine, such as (please tick the appropriate ones)
[ ] Urticaria [ ] Angioedema [ ] Shortness of breath [ ] Rhinitis [ ] Watery eyes [ ] Anaphylactic shock [ ] None of the following

Do you feel apprehensive about the second dose of vaccination against SARS-CoV-2 ?
[ ] Yes, greater than before the first dose of the vaccine, ...(please specify)
[] Yes, smaller than before the first dose of the vaccine
[] No

Did you have a test for SARS-CoV-2 infection between the first and second doses of the vaccine?
[ ] YES, the result was positive [ ] YES, the result was negative [ ] NO
