# Supplementary material for: Before and After: Attitude and Adverse Effects Induced by the First and Second Doses of mRNA BNT162b2 Vaccine among Healthcare Professionals in the First Weeks after Their Introduction in Poland
Source: Vaccines (Basel). 2023 Apr 22;11(5):883. doi: 10.3390/vaccines11050883 (PMC10222834; doi:10.3390/vaccines11050883)
Supplement: Supplementary file 1 [file vaccines-11-00883-s001.zip › Vaccines-2278513-Supplementary File S3.pdf]

QUESTIONNAIRE FOR A PATIENT WHO HAS RECEIVED TWO DOSES OF mRNA VACCINATION against COVID-19

AGE:
GENDER: female, male
Place of residence:
[ ] village
[] city of up to 50,000 inhabitants [ ] city of 50,000 to 100,000 inhabitants [ ] city of 100,000 to 500,000 inhabitants [ ] city of more than 500,000 inhabitants
Education: ....
Are you a health care professional? [ ] YES [ ] NO
Occupation: doctor, nurse, paramedic, orderly, administrative worker, technical worker (please tick the appropriate one)

Do you have a history of SARS-CoV-2 infection?
[ ] YES, confirmed by molecular testing (PCR) or antigen test
[] I had/have typical symptoms of SARS-CoV-2 infection, but without confirmation by laboratory tests
[] I have a positive titer of anti-CoV IgG antibodies and have had symptoms of infection
[] I have a positive titer of anti-CoV IgG antibodies, but have had no symptoms of infection
[] NO

If you have experienced SARS-CoV-2 infection, please tick the symptoms you experienced during the infection:
[ ] fever
[] weakness
[ ] chills
[] muscle/joint pain
[] headache
[] cough
[ ] loss of smell
[] olfactory disorders such as abnormal olfactory sensations/unpleasant odours
[] loss of taste
[] abdominal pain
[] nausea, vomiting
[] diarrhoea
[] other .... (please specify)

Did you experience SARS-CoV-2 infection after receiving the 1st dose of vaccine?
[ ] YES, confirmed by molecular testing (PCR) or antigen test, ... (number of days after the 1st dose)
[] NO

Did you experience any adverse reactions after receiving the first and second doses of the vaccine?
[ ] Yes [ ] No

After the 1st dose:

SYMPTOM	YES	NO	DURATION
pain at the injection site			
swelling at the injection site			
redness at the injection site			
pruritus at the injection site			
fatigue			
poor well-being			
shivers			
fever			
muscle/joint pain			
nausea/vomiting			
headache			
diarrhea			

lymph node enlargement			
insomnia			
acute peripheral facial nerve paralysis			
allergic reaction			
anaphylaxis / anaphylactic shock requiring adrenaline administration			
olfactory disorder			
taste sensation disorders			
others			

After the 2nd dose:

SYMPTOM	YES	NO	DURATION
pain at the injection site			
swelling at the injection site			
redness at the injection site			
pruritus at the injection site			
fatigue			
poor well-being			

shivers			
fever			
muscle/joint pain			
nausea/vomiting			
headache			
diarrhea			
lymph node enlargement			
insomnia			
acute peripheral facial nerve paralysis			
allergic reaction			
anaphylaxis / anaphylactic shock requiring adrenaline administration			
olfactory disorder			
taste sensation disorders			
others			

Please fill in only if you are a recovering patient:
In your opinion, were the adverse reactions after vaccination similar to those during SARS-CoV-2 infection?

[ ] YES [ ] NO

Have you also previously had post-vaccination reactions to other vaccinations?

[ ] YES [ ] NO

Please indicate which vaccines were followed by adverse reactions:
influenza pneumococcus menongococcus tetanus hepatitis A hepatitis B

Please indicate which post-vaccination reactions have occurred:

- pain at the injection site
- redness + swelling
- headache
- shivers
- feeling of breakdown
- muscle pains
- fever
- enlargement of lymph nodes
- facial nerve paralysis
- rash
- swelling of the lips and tongue
- anaphylactic shock

Do you supplement with Vit. D, If yes, in what dose?

- regularly
- occasionally
- no

Do you have a chronic illness? If yes, please tick which one:

- hypertension
- type 1 diabetes
- type 2 diabetes
- obesity

- POCHP
- recurrent respiratory tract infections
- bronchial asthma
- heart diseases (which ones)
- cancer (which one)
